# Brain Injury Differences in Frontal Impact Crash Using Different Simulation Strategies

**DOI:** 10.1155/2015/348947

**Published:** 2015-10-01

**Authors:** Dao Li, Chunsheng Ma, Ming Shen, Peiyu Li, Jinhuan Zhang

**Affiliations:** ^1^State Key Laboratory of Automotive Safety and Energy, Department of Automotive Engineering, Tsinghua University, Beijing 100084, China; ^2^Bioengineering Center, Wayne State University, Detroit, MI 48201, USA

## Abstract

In the real world crashes, brain injury is one of the leading causes of deaths. Using isolated human head finite element (FE) model to study the brain injury patterns and metrics has been a simplified methodology widely adopted, since it costs significantly lower computation resources than a whole human body model does. However, the degree of precision of this simplification remains questionable. This study compared these two kinds of methods: (1) using a whole human body model carried on the sled model and (2) using an isolated head model with prescribed head motions, to study the brain injury. The distribution of the von Mises stress (VMS), maximum principal strain (MPS), and cumulative strain damage measure (CSDM) was used to compare the two methods. The results showed that the VMS of brain mainly concentrated at the lower cerebrum and occipitotemporal region close to the cerebellum. The isolated head modelling strategy predicted higher levels of MPS and CSDM 5%, while the difference is small in CSDM 10% comparison. It suggests that isolated head model may not equivalently reflect the strain levels below the 10% compared to the whole human body model.

## 1. Introduction

### 1.1. Finite Element Modelling of Brain Injury

Head trauma is a main type of injuries seen in the motor vehicle crashes. It is estimated by the World Health Organization that the head injuries account for half of the 1.3 million annual deaths and the 50 million injuries caused by traffic accidents worldwide [1]. FE modelling has been a well-developed approach to investigate brain injuries. A number of human FE models have been developed in detail so as to investigate the injury mechanism and predict the injury risk in real world crash scenarios [2–4]. Due to the huge computational consumption of the whole human body model, some isolated models extracted from the whole human body model were used. For example, the isolated head model extracted from the Total Human Model for Safety (THUMS) has been used to study brain injuries of football players [5]. This method is a feasible and high-efficient way to study the brain injury mechanism by replicating the head motions. However, there are few studies which compared the differences between these two FE simulation strategies in detail: (1) using the whole human body model and (2) using the isolated head model from the same whole human body model, with the head motions from (1) as the inputs. If the second method is proved to be an equivalent alternative approach, the FE injury analysis and further restraint system optimization could be easier using this reasonable simplification. Otherwise, this simplification could only be treated as an approximation with limitations and should be employed with caution.

### 1.2. Measurements of Brain Injury

For quantitative comparison of the aforementioned strategies, proper injury measurements need to be selected. Head Injury Criterion (HIC) was first reviewed. It was introduced as a fail/pass criterion of head injuries in 1972 as specified in the Federal Motor Vehicle Safety Standard Number 208 (FMVSS 208). During the past decade, HIC has been widely used in assessment of head injury in the cadaver test and dummy test, since it is a good predictor of skull fracture. However, researches have revealed that the risk of the occupant who sustained a brain injury without skull fracture would be greater than the ones who sustained a skull fracture without a brain injury (3.83% and 0.05%, resp.) [6]. That means more attention should be paid to brain tissue damage (such as concussion, contusion, hematoma, etc.) in motor vehicle frontal impacts. Meanwhile, the statistics shows that the incident rate of Traumatic Brain Injury (TBI) has increased since 1995 for emergency departments and outpatient departments visits [7].

Due to the capability limitation of the HIC, stress and strain metrics such as VMS, MPS, and CSDM were used to study the intracranial stress development and distribution [8–10]. CSDM that aims to evaluate cumulative damage of brain tissue was developed based on the theory that the overstretched axons remain in dysfunction. It is defined as the volume percentage of the brain that ever exceeds the various specified first principal strain threshold during the impact period. Since it conveys the information of all overstretched elements in the progress, it may predict better than maximum stress or strain levels at a particular time point. Additionally, several metrics based the rotational kinematics were also built to study the mechanism of brain injuries [11, 12]. However, these evaluation methods can only be employed in the condition of the head kinematics acquired before.

Based on the above discussions, VMS, MPS, and CSDM will be adopted as the measurements to compare the detailed responses of the two different modelling strategies in Front Rigid Barrier (FRB) test and 40% Offset Deformable Barrier (ODB) test, respectively. THUMS will be taken as a good example of whole body human model with validated head and brain submodel.

## 2. Methods

### 2.1. Whole Human Body FE Model

The THUMS version 4 AM50 is a validated human body FE model representing a mid-sized male (175 cm and 77 kg) including the complex organs and tissues of the human body [2]. The two kinds of simulation strategies were performed based on THUMS.

**Figure 1:**
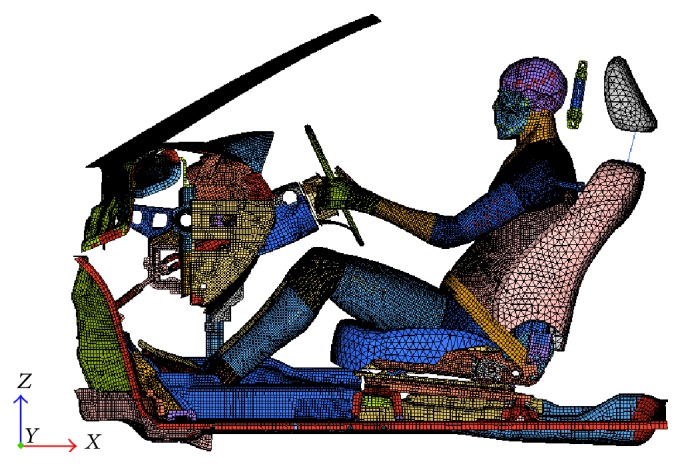
Sled model carrying THUMS.

The THUMS seated on the sled FE model was used to reconstruct the crash experiments, which were 56 km/h FRB test and 64 km/h 40% ODB test. The sled FE model of a car was provided by Guangzhou Automobile Group Co., Ltd., and was validated against data from 56 km/h Front. The sled model replicated the vehicle kinematics from the full car crash simulation and therefore can be used to integrate with the whole THUMS for injury study. Meanwhile, the restraint system was modeled to reflect the real conditions. A load limit of the retractor of 2.25 kN was used and the friction coefficients of both slip-rings were set to 0.2, according to the restraint system designs in the realistic crash test. The sled model which carries the restrained THUMS whole human body model is shown in Figure 1.

### 2.2. Isolated Human Head FE Model

**Figure 2:**
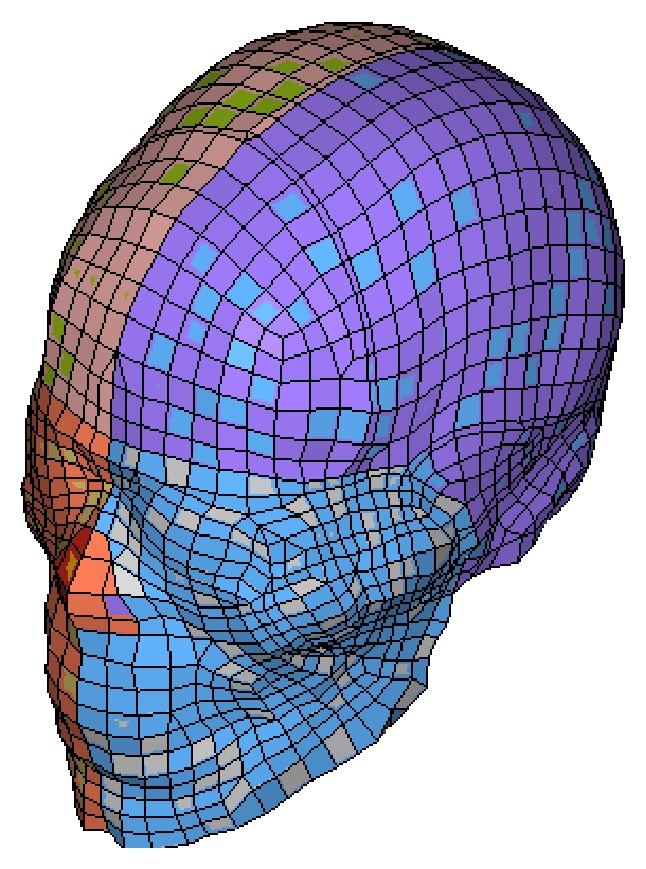
Isolated head model.

The isolated human head FE model was extracted from the THUMS (see Figure 2). Its brain consists of hexahedral solid elements representing the cerebrum, cerebellum, brainstem with distinct white and gray mater, and cerebral spinal fluid (CSF). The head model was validated with the cadaver tests on translational and rotational impact [13, 14]. A detailed validation of THUMS head-neck submodel could be found in literature [15].

**Figure 3:**
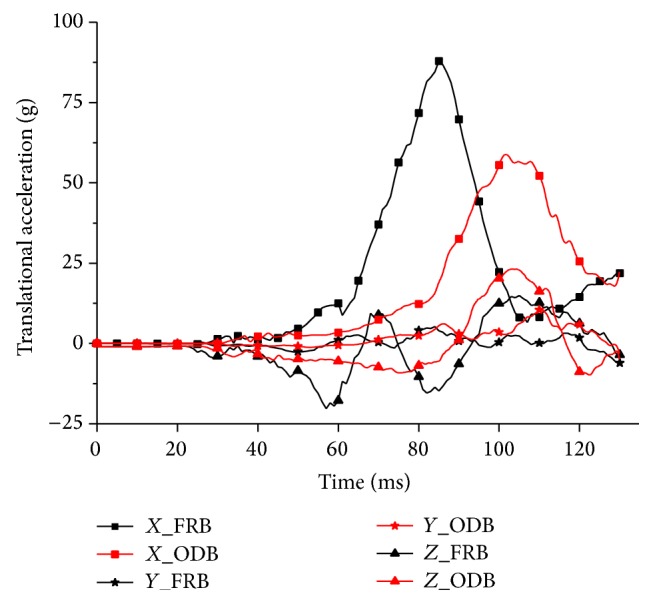
Translational acceleration inputs for isolated head model simulation.

**Figure 4:**
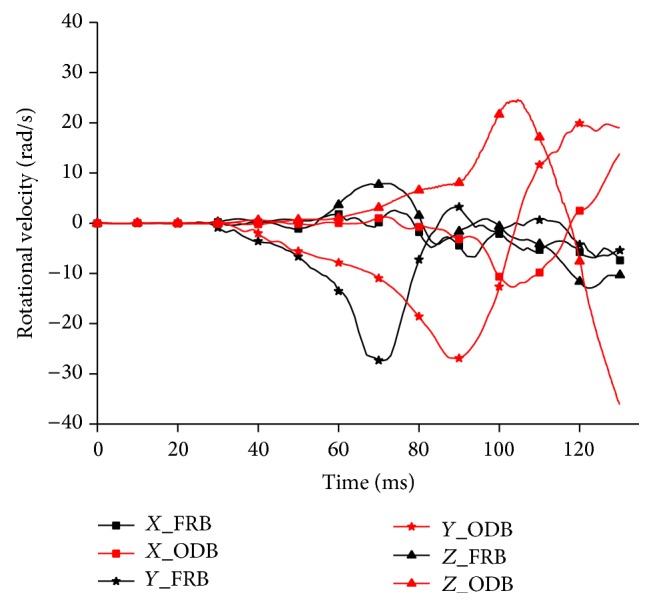
Rotational velocity inputs for isolated head model simulation.

The skull in this FE model was modified as a rigid body to apply the given motions at six Degrees of Freedom (DOF), and this methodology was commonly adopted in brain injury FE studies [12, 16, 17]. Translational and rotational kinematics was measured at the center of gravity (CG) of the head using the constrained interpolation method [18]. This method would acquire the kinematics of the dependent node (e.g., CG) from the interpolation of the surrounding independent nodes. Subsequently, the linear accelerations and angular velocities measured in sled simulation (using whole human body model) were input to the simulation using isolated head model as the prescribed boundary conditions to simplify the reconstruction (Figures 3 and 4).

### 2.3. Brain Injury Metrics

The spatial distribution of VMS and MPS was analyzed first. The VMS and MPS of the brain would reflect the internal stress/strain concentrations and vulnerable spots by using each modelling strategy. Since some other regions without the highest strain level may be overlooked because the stress/strain level drops to normal after the damage, the cumulative measurement CSDM will cover that additional information. Animal experiments [19] showed loss of axonal transport may occur at a strain level of 15% and 18%. The occupants studied in the sled simulations were belted with the airbag deployed normally; thus it is reasonable to not expect severe brain injury. In this study, lower strain thresholds of 5% and 10% for CSDM were chosen to demonstrate the difference in two kinds of collision situations, although low risks of lethal injury were associated with these strain levels.

**Figure 5:**
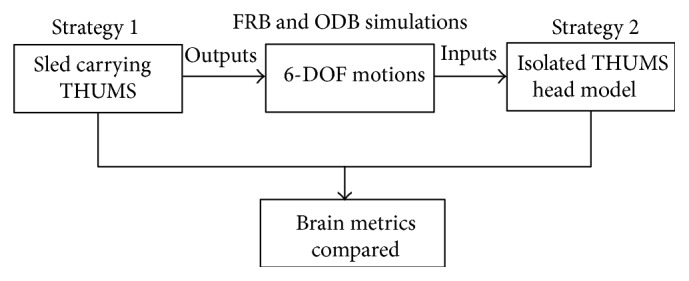
Procedures of the modelling strategies.

With the brain response from the whole THUMS body simulations, the brain response from the isolated THUMS head could be compared. The logic diagram of the simulations is shown in Figure 5.

## 3. Results

The difference of these two modelling strategies lies in the different boundary conditions. The neck would transfer the load which comes from the body when using the whole THUMS, while the brainstem, as one of the channels to transfer the load, would not work in the isolated head modelling strategy. Therefore, the cerebrum is mainly discussed in the next two parts.

### 3.1. VMS Distributions

**Figure 6:**
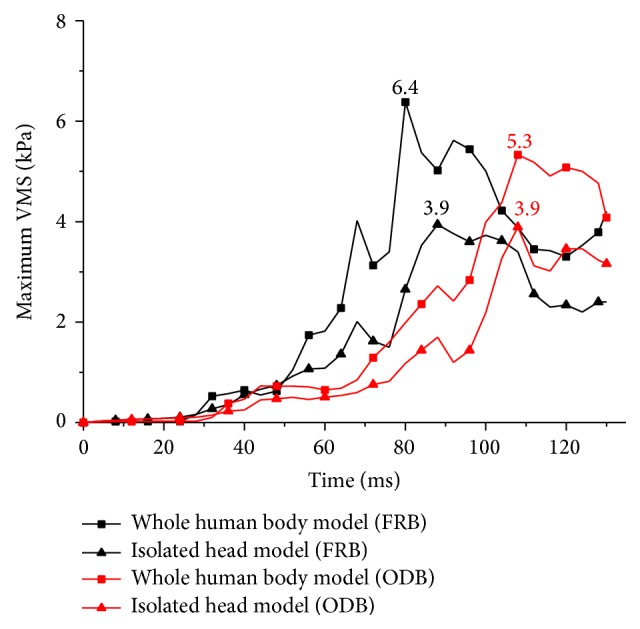
Maximum VMS in the cerebrum.

Figure 6 presents the time histories of the maximum VMS in cerebrum. It shows that the maximum VMS in whole human body models are both higher than that in isolated head models. The VMS gotten from the FRB simulations (black curves) start to increase rapidly after 50 ms and reach their own maximum value at 80 and 88 ms, respectively. The red curves represent the VMS in ODB simulations beginning to increase around 70 ms and reaching their maximum value at 108 ms simultaneously, while the isolated head model gets its maximum value later than the whole human body model in FRB simulations.

**Figure 7:**
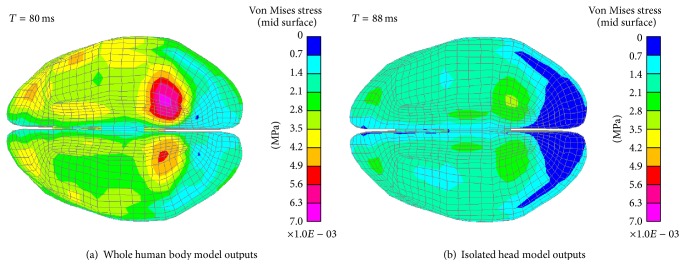
VMS distribution of the cerebrum in FRB simulations (bottom view).

Figure 7 shows the distribution of the VMS in the FRB simulations. The maximum VMS in each case using different modelling strategies is 6.4 kPa and 3.9 kPa, respectively. The large VMS of the whole human body model concentrate at the occipitotemporal region close to the cerebellum. The distribution pattern of the VMS in the isolated head model is similar to that of the whole human body model. However, the magnitude of the VMS is lower than that of the whole human body model.

**Figure 8:**
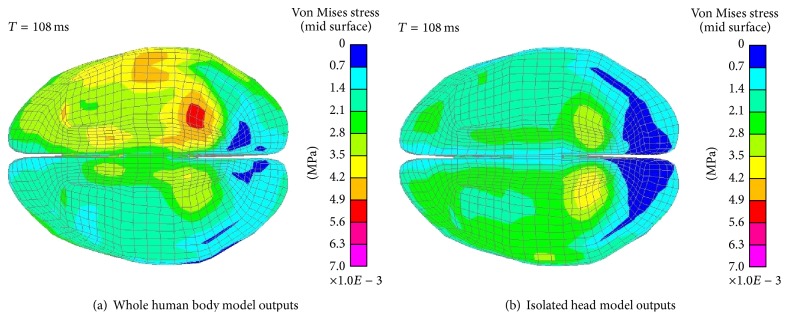
VMS distribution of the cerebrum in ODB simulations (bottom view).

Figure 8 shows the distribution of the VMS at 108 ms in ODB simulations. The maximum VMS in the two modelling strategies are 5.3 kPa and 3.9 kPa, respectively. The maximum VMS concentrate at the lower cerebrum and occipitotemporal region, which is similar to the results of the FRB simulations. The magnitudes of the VMS in ODB simulations are lower than those in FRB simulations. This is caused by the lower longitudinal (*x*-axis) deceleration in ODB crashes compared to FRB crashes.

It is found that the distributions of the VMS are close in different collision simulations. The maximum VMS in the whole human body model are both higher than that in the isolated head model regardless of the different collision simulations. And the VMS mainly concentrate at the occipitotemporal region.

### 3.2. MPS Distributions

**Figure 9:**
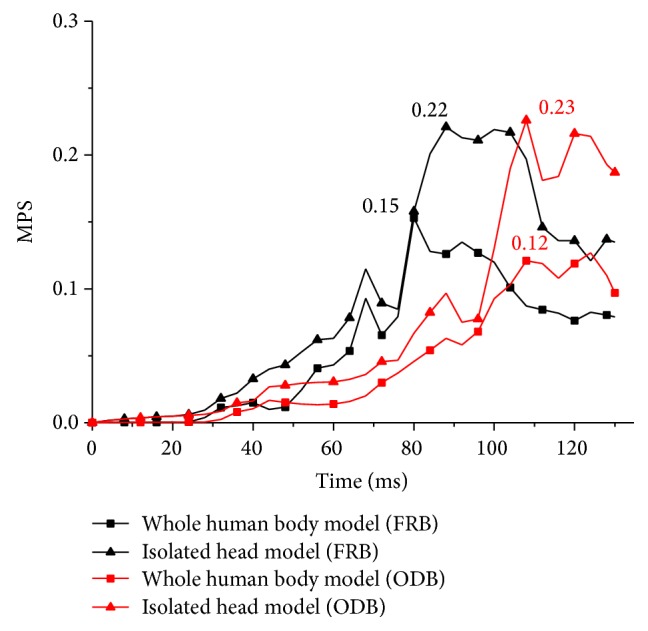
MPS in the cerebrum.

Figure 9 presents the time histories of the MPS in cerebrum by using different modelling strategies. It is clearly shown that the MPS in the isolated head models represented by the black curves are higher than that in the whole human body models. The MPS reach their maximum value in ODB simulations at the same time, while, in FRB simulations, the isolated head model reaches its maximum value later than the whole human body model, which has the same trend with the VMS.

**Figure 10:**
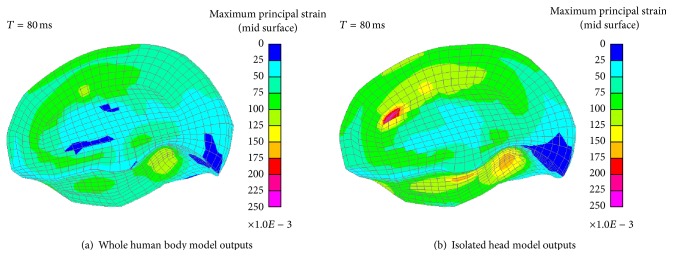
MPS distribution of the right brain in FRB simulations.

In the FRB simulation, the head of the THUMS impacts the center of the airbag and then rebounds back normally.  Figure 10 is the MPS distribution of the right brain in FRB simulations. The MPS of the two modelling strategies are 0.15 and 0.22, respectively. It shows that the MPS appears at the spots near the corpus callosum and the large MPS mainly concentrate upon the region between the two half cerebrums and the occipitotemporal region close to the cerebellum.

**Figure 11:**
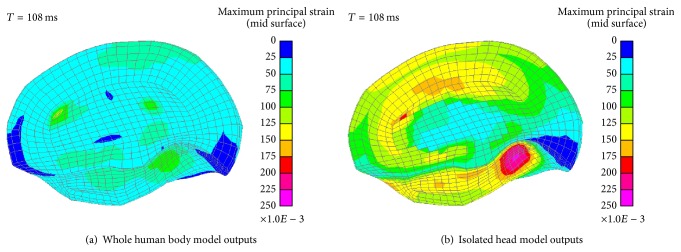
MPS distribution of the right brain in ODB simulations.

Figure 11 shows the MPS distribution of the right brain at 108 ms in ODB simulations. The MPS of these two simulations are 0.12 and 0.23, respectively. Their distribution areas are very similar to that in the FRB simulations. And the MPS mainly concentrate upon the occipitotemporal region close to the cerebellum.

In the FRB simulations, the head only had large rotation around the lateral direction (*y*-axis) regardless of the minor rotation of others, whereas, in the ODB simulation, the head would rotate around the vertical direction (*z*-axis) positively after it impacts the airbag. The large strain at the upper cerebrum may be caused by the head rotations at the *x*-axis and *z*-axis since these two relative motions would induce more severe contact force of the two half cerebrums.

The isolated head models get lower VMS and higher MPS according to the results shown above. VMS is a kind of equivalent stress calculated from the three principal stresses. Higher MPS means higher first principal stress normally, while the value discrepancy between VMS and MPS shows that the second/third principal stress based on the second/third principal strain may also have a great influence on the VMS value.

### 3.3. CSDM

**Figure 12:**
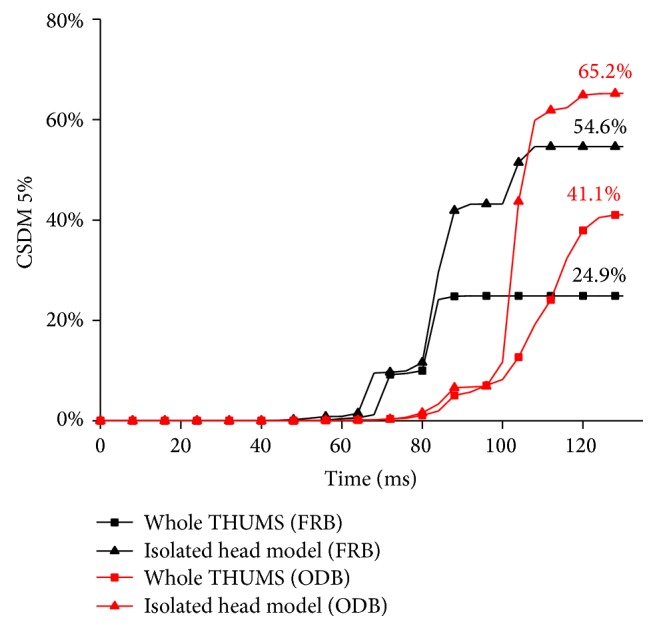
Comparison of the CSDM 5% in different modelling strategies.

Figure 12 shows the time history of CSDM 5% by using different modelling strategies. Black curves represent the two modelling strategies in the FRB simulations beginning to increase rapidly after 80 ms along the whole simulations, which are 20 ms earlier than the red curves. The ultimate CSDM 5% values of the whole human body model and isolated head model calculated from the FRB simulations are 24.9% and 54.6%, respectively. And the ultimate CSDM 5% values from the ODB simulations represented by the red curves are 41.1% and 65.2%, respectively.

The isolated head model inputted with THUMS kinematics in FRB simulation reaches its maximum CSDM 5% of 54.6%, which is 29.7% higher than that in the whole human body modelling strategy, while the isolated head model in ODB simulation reaches its maximum CSDM 5% of 65.2%, which is also 24.1% larger than that from the whole human body modelling strategy. Both CSDM 5% values by using isolated head modelling strategy are higher than the whole human body model, in both collision situations of FRB and ODB.

**Figure 13:**
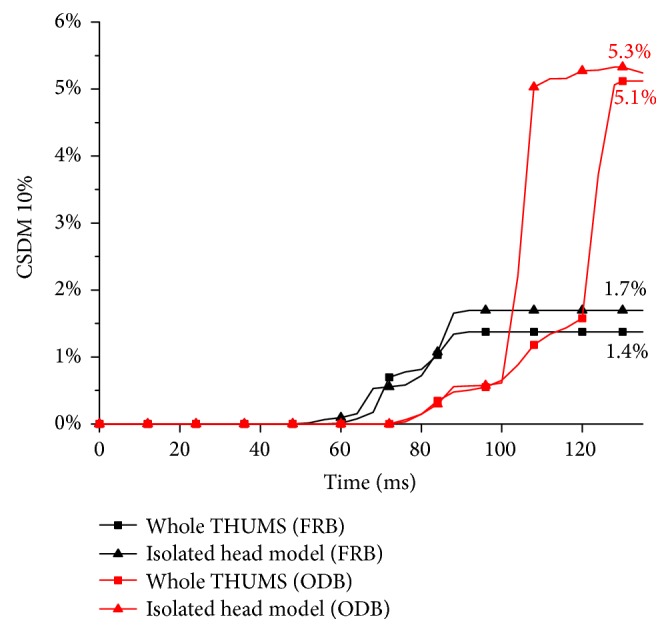
Comparison of the CSDM 10% in different modelling strategies.

Figure 13 shows the time histories of CSDM 10% using different modelling strategies. The trends of the CSDM 10% curves are similar to the CSDM 5% curves. The whole human body model in FRB simulation reaches its maximum CSDM 10% of 1.4%, while the maximum CSDM 10% of the two isolated head models is 1.7% accordingly. In the ODB simulations, the whole human body model and the isolated head model reach their maximum CSDM 10% of 5.1% and 5.3%, respectively.

The outputs using isolated head modelling strategy are also the highest. However, the difference between the two modelling strategies in the two collision situations is decreased by only 0.2%-0.3%. Compared to the trends in the CSDM 5%, it is found that the differences of elements strain are mainly between the 5% and 10%. It also means that the elements strain larger than 10% would not be much different. Since the CSDM difference is concentrated below the 10% values, it suggests that using the isolated head modelling strategy to study the brain injuries might predict higher strains levels than the whole human body modelling strategy.

## 4. Discussion

This study used two models, whole body human model and isolated head model with prescribed motions, to predict the brain injury in frontal impacts. The former method replicated the real crash scene well. However, the enormous amount of elements (mainly from the whole human body model) requires a longer computing time and more computing resources. The method of using isolated head model has been widely used to study the brain injury metrics assumedly as an alternative approach. It can reflect the motion of the head with the benefit of its simplicity and time-saving. The errors were analyzed quantitatively in the aforementioned simulation studies.

There are three possible reasons that may cause this discrepancy. First, the cutting-off of the brain stem from the spinal cord makes the boundaries not identical. Normally free surface will be found in simplified isolated head model. In realistic case, this consecutiveness makes the brain have a soft boundary near the Foramen magnum, which is hard to mimic in isolated head modelling strategy. Second, the data processing may produce errors caused by output rate and simulation time steps. The accuracy of motion output of whole body simulation depends on the frequency of output. This error may further become the input error in isolated head model. Third, the simplification of making the skull as rigid part rather than the elastic/plastic bone material in the whole human body model may also matter. This effect is minor due to the large elasticity difference between the cerebral tissue and the adjacent skull. However, the local stress may be affected by this stiffer boundary in isolated head modelling strategy. Further investigation could be conducted by parametric studies.

Due to the protection of the belt and the airbag, the impact circumstances (FRB and ODB) would not cause too much brain damage to the occupants. So it can only cause lower level stain during the impact and it may need to apply more severe impact loadings to address this difference better.

## 5. Conclusions

The following three specific conclusions can be drawn from the investigation of brain response simulations for head impacts using whole body human model and isolated head FE models:

- The maximum VMS of the isolated head models are 2.5 kPa and 1.4 kPa lower than those in the simulations using whole body model. And the stress distribution patterns are similar, mainly concentrated at the lower cerebrum and occipitotemporal region close to the cerebellum.
- The MPS of the isolated head model are higher than the whole human body model regardless of being in FRB simulations or ODB simulations.
- The isolated head models predicted 24.1% higher value of the CSDM 5% in FRB simulations and 29.5% higher value of the CSDM 5% in ODB simulations than the whole human body models do. But the difference in CSDM 10% level is smaller.
- The strain difference between the two modelling strategies mainly lies in the CSDM level below 10%, since the loading conditions are not very intense in this study.
- Based on the findings of this study, it is suggested that, for highly accurate analysis of brain injury or study of the region around the brain stem, the whole body human model is recommended. The simplified isolated head model could be used for overall injury trend discussion carefully with limitations.
